# Interleukin-1 Beta in Peripheral Blood Mononuclear Cell Lysates as a Longitudinal Biomarker of Response to Antidepressants: A Pilot Study

**DOI:** 10.3389/fpsyt.2021.801738

**Published:** 2021-12-24

**Authors:** Panagiotis Ferentinos, Eirini Maratou, Anastasia Antoniou, Alessandro Serretti, Nikolaos Smyrnis, Paraskevi Moutsatsou

**Affiliations:** ^1^2nd Department of Psychiatry, “Attikon” University General Hospital, National and Kapodistrian University of Athens, Athens, Greece; ^2^Department of Clinical Biochemistry, “Attikon” University General Hospital, National and Kapodistrian University of Athens, Athens, Greece; ^3^Department of Biomedical and Neuromotor Sciences, University of Bologna, Bologna, Italy

**Keywords:** antidepressants, Interleukin-1 beta, lysates, major depressive disorder, monocytes, response

## Abstract

Interleukin-1 beta (IL1β) is primarily produced by monocytes in the periphery and the brain. Yet, IL1β protein levels have to date been investigated in major depressive disorder (MDD) and antidepressant response using either plasma or serum assays although with contradictory results, while mononuclear cell assays are lacking despite their extensive use in other contexts. In this pilot study, we comparatively assessed IL1β in mononuclear lysates and plasma in depressed MDD patients over treatment and healthy controls (HC). We recruited 31 consecutive adult MDD inpatients and 25 HC matched on age, sex, and BMI. Twenty-six patients completed an 8-week follow-up under treatment. IL1β was measured in both lysates and plasma in patients at baseline (T0) and at study end (T1) as well as in HC. We calculated ΔIL1β(%) for both lysates and plasma as IL1β percent changes from T0 to T1. Seventeen patients (65.4% of completers) were responders at T1 and had lower baseline BMI than non-responders (*p* = 0.029). Baseline IL1β from either plasma or lysates could not efficiently discriminate between depressed patients and HC, or between responders and non-responders. However, the two response groups displayed contrasting IL1β trajectories in lysates but not in plasma assays (response group by time interactions, *p* = 0.005 and 0.96, respectively). ΔIL1β(%) in lysates predicted response (*p* = 0.025, AUC = 0.81; accuracy = 84.6%) outperforming ΔIL1β(%) in plasma (*p* = 0.77, AUC=0.52) and was robust to adjusting for BMI. In conclusion, ΔIL1β(%) in mononuclear lysates may be a longitudinal biomarker of antidepressant response, potentially helpful in avoiding untimely switching of antidepressants, thereby warranting further investigation.

## Introduction

Major depressive disorder (MDD) is a severe psychiatric disease with lifetime prevalence in excess of 15% and the second leading cause of disability worldwide (1). At present, MDD diagnosis and treatment are based on subjective assessment of symptoms. Antidepressant response rates range from less than one third to more than two thirds depending on setting and patient characteristics (2, 3). In naturalistic studies, up to one third achieve remission (4) and up to one half will relapse within a year (5).

The pathophysiology of MDD implicates both reduced neurogenesis and increased inflammation (6, 7). Peripheral inflammation has been observed in subsets but not all MDD patients (8, 9). Several studies have highlighted the importance of various proinflammatory cytokines in the pathophysiology of MDD and antidepressant response, with TNFα, interleukin 1 beta (IL1β), IL4, IL6, IL8, and IL10 (10–16) having a most prominent role.

IL1β is a key mediator of inflammatory response and also involved in cell proliferation, differentiation, and apoptosis (17, 18). In the periphery, IL1β is primarily produced by cells of the mononuclear phagocytic lineage, but also by numerous other cell types (19). In the brain, IL1β is expressed in the hypothalamus, hippocampus, cerebral cortex, and thalamus; it is secreted by astrocytes, oligodendrocytes, neurons, and microglia (brain macrophages) in response to inflammatory stimuli (20, 21). Moreover, circulating IL1β produced in response to peripheral stimuli can cross the blood–brain barrier via saturable transport resulting in peripheral inflammation affecting the brain (22).

IL1β is strongly involved in the pathophysiology of MDD mainly in the context of the inflammasome hypothesis (23). NLRP3, also known as inflammasome, is a cytosolic protein complex forming in response to pathogens, which activates caspase-1 resulting in cleaving of the pro-IL1β to biologically active, secreted IL1β (24). The inflammasome hypothesis of depression states that psychological stress activates NLRP3 and proposes the pathway between NLRP3 to IL1β as an underlying mechanism of MDD (23). Furthermore, IL1β activates the HPA axis and can lead to glucocorticoid receptors functional resistance, a mechanism widely investigated in the relation between inflammation and depression (25, 26). Finally, IL1β has been directly associated with decreased neurogenesis in human hippocampal progenitor cells by affecting the availability of tryptophan and by upregulating enzymes of the neurotoxic arm of the kynurenine pathway (27).

To investigate the role of IL1β in MDD and antidepressant response, several questions need to be answered: (1) is there a significant difference in the circulating levels of the cytokine among patients with MDD and healthy people, (2) does the severity of depression correlate with IL1β levels, (3) is there any significant difference between baseline IL1β levels of antidepressant responders vs. non-responders and could it be used as a prognostic marker of response to treatment, (4) is there any effect of antidepressant treatment on circulating IL1β independent of response status, and finally, (5) is there a distinct pattern of variation of IL1β levels over time in responders and non-responders and could this discern among them?

These questions have been addressed in several previous studies; however, their findings are often contradictory and finally non-conclusive and recent meta-analyses often provide non-significant results (10, 12, 14, 16, 28, 29). These contradictory results could have been caused by between-study heterogeneity. Across various studies, inclusion criteria were inconsistent, patient characteristics, or duration of follow-up varied, while IL1β levels were measured in plasma in some studies or serum in others (10, 16).

Although IL1β is mainly produced by monocytes in the periphery, which, when activated, infiltrate the brain and interact with microglial cells contributing to the pathophysiology of MDD (30), IL1β protein levels have as yet been investigated in MDD using either plasma or serum assays; mononuclear cell assays have been used in MDD only in transcriptional studies to measure IL1β mRNA (31–34). However, mononuclear cells have been extensively used in other contexts to measure IL1β protein levels (35, 36). Mononuclear cells present an acceptable model for the simulation of the brain since significant gene expression similarities have been found between blood and brain (37–40), including several candidate genes for mood disorders (41, 42). Therefore, we hypothesized that IL1β derived from mononuclear lysates might better discern MDD patients from controls compared with plasma IL1β and that baseline lysate IL1β levels or their changes over antidepressant treatment might more efficiently discriminate responders from non-responders.

In this pilot study, we hence aimed to investigate the role of IL1β in MDD and antidepressant response by comparatively assessing it in mononuclear lysates and plasma assays in depressed MDD patients over treatment and healthy controls. Specifically, we aimed to investigate first, how IL1β assays in plasma or lysates compare in their ability to discriminate between depressed MDD patients and controls, as well as between future responders and non-responders; second, whether depression severity correlates with plasma or lysate IL1β levels; and finally, we sought to assess whether longitudinal changes in IL1β concentrations derived from plasma or lysates would differ in their ability to reflect patients' response status.

## Materials and Methods

### Participants

Consecutive adult inpatients with a DSM-5 diagnosis of MDD hospitalized due to a major depressive episode (MDE) of at least moderate severity were recruited during an 18-month period (2018–2019) for the current study. Patients were excluded if they had been diagnosed with intellectual disability, severe personality disorder, a serious neurological or medical disease, or had a history of substance or alcohol misuse in the past 6 months.

Patients were treated in an open-label manner by their physician using antidepressants within the recommended dose range, antidepressant combinations, and augmentation with atypical antipsychotics or lithium. They were followed up over an 8-week period, initially within the inpatient ward and subsequently on an outpatient basis. Any modification of medication regime was allowed as judged appropriate during the follow-up period.

Healthy controls with no psychiatric history, matched to patients for age, BMI, and sex distribution, were also recruited by convenience sampling. They were either members of the staff or caregivers of patients attending other medical clinics. Exclusion criteria for controls were the same as for patients.

All participants provided written informed consent before being included in the study, which was carried out in accordance with the Helsinki declaration and was approved by the local Research Ethics Committee.

### Clinical Assessment

Patients' current and lifetime diagnosis was established with SCID-5 (43). Sociodemographic characteristics were recorded while clinical features were extracted from patients' interviews, primary caregivers, and medical records. Medical comorbidity, with a special emphasis on cardiometabolic diseases (e.g., diabetes mellitus, hypertension, dyslipidemia), was similarly recorded using the Cumulative Illness Rating Scale (CIRS) and the total score of CIRS items 1–13 was calculated (44). Patients' clinical state was assessed at baseline (T0) and at end of study (T1, i.e., 8 weeks post-baseline) with the Montgomery–Åsberg Depression Rating Scale (MADRS), comprising 10 items rated 0–6 (45). Change in MADRS scores between these two time-points as a percentage of baseline MADRS scores was calculated for every patient: ΔMADRS(%) = [MADRS (T0) – MADRS (T1)]/MADRS (T0) × 100; therefore, a positive ΔMADRS(%) denoted an improvement (decrease) of depression severity. Patients with a ΔMADRS(%)≥50 were considered responders. Fluoxetine equivalent dose of antidepressants administered was calculated for each patient (46).

Healthy controls were assessed with a brief clinical interview including items on demographic data, personal history, any present complaints, psychiatric and medical history, past and current medical or psychiatric therapies, and a brief mental state examination.

### Plasma and Lysate Assays

Patients at T0 and T1 and healthy controls were subjected to morning (8 a.m.) blood sampling after overnight fasting. Upon punctuation, 1 ml of blood was centrifuged at 3,000 × g, 5 min at 22°C, and plasma was collected and kept at −80°C, until the determination of IL1β plasma levels. The rest of the blood was diluted 1/1 (v/v) with phosphate buffer saline (PBS, without Ca^2+^, Mg^2+^), placed on Histopaque-1077 (1/2, v/v) and centrifuged at 400 × *g* for 30 min at 22°C, to isolate the “buffy” coat (mononuclear inter-phase layer). Mononuclear cells were then resuspended in PBS and washed twice (150 × g, 10 min at 22°C). Erythrocytes were lysed with BD Pharm Lyse (lysing reagent; BD Biosciences Pharmingen, San Jose, CA, USA). The exact number of cells was determined cytometrically with Flow-Count Flurospheres (Beckman-Coulter, Miami, USA). Mononuclear cells were then lysed by PathScan Sandwich ELISA lysis buffer enriched with PMSF 1 mM, incubated for 15 min, on ice and then centrifuged at 14,000 × g, 5 min at 4°C. The lysates were aliquoted and kept at −80°C, until the protein quantification. Protein levels in the lysates were determined by Coomasie Plus Bradford assay kit (Pierce Biotechnology, Inc. USA).

The levels of IL1β in mononuclear cell lysates were determined by ELISA kit (Mabtech, Inc., Cincinnati, USA) with sensitivity 0.316 pg/ml, intra-assay variation 1.9%, and inter-assay variation 12.4%. In each well, 45 μg of total protein was loaded, to normalize and produce comparable results. The plasma levels of IL1β were determined by a commercially available kit (Human IL1β high sensitivity ELISA kit; Invitrogen, Thermo Fisher Scientific Inc.) with sensitivity 0.05 pg/ml, intra-assay 6.7%, and inter-assay coefficient of variation 8.1%. The usage of different ELISA kits for the lysates and the plasma samples was implemented due to extremely low levels of the cytokine in the blood of subjects with low or no peripheral inflammation.

For both plasma and lysate assays, change in IL1β concentrations between T0 and T1 as a percentage of baseline IL1β concentrations was calculated for every patient: ΔIL1β (%) = [IL1β (T0) – IL1β (T1)]/IL1β (T0) × 100; therefore, a positive ΔIL1β (%) denoted that IL1β concentrations decreased.

### Data Analyses

Statistical analyses were carried out with STATA 14.0. The sample's clinicodemographic characteristics, treatment-related parameters, and laboratory assays were explored with descriptive statistics; normality of continuous variables was checked with the Shapiro–Wilk test. Group comparisons were performed with χ^2^ or Fisher's exact test, independent samples *t*-test, or Mann–Whitney test, as appropriate. Relationships among variables were explored with Spearman correlations or linear regressions. Power analyses implemented with G^*^Power 3.1.9.7 calculated the minimum effect size detectable with adequate power.

Variations of IL1β concentrations over time by response status were investigated in linear mixed models, with response group, time, and their interaction along with potential confounders as fixed effects and subjects as random effect; IL1β concentrations were log-transformed before analysis due to non-normality. A significant response group by time interaction would suggest response specific IL1β trajectories.

Finally, we investigated whether response status could be predicted by baseline IL1β concentrations or ΔIL1β (%) (from lysates or plasma) in logistic regression models, adjusting for potential confounders. Relevant receiver operating characteristic (ROC) curves were produced and compared. Classification achieved at the optimal probability threshold was then assessed with diagnostic metrics: sensitivity (Sn), specificity (Sp), positive likelihood ratio (PLR), negative likelihood ratio (NLR), positive predictive value (PPV), negative predictive value (NPV), and accuracy or correct classification rate (CCR).

## Results

We initially recruited 31 depressed patients (48.4% females, aged 53.0 ± 9.6 years, BMI 27.6 ± 4.9) and 25 healthy controls (HC) for the current study. Five patients were lost during follow-up (drop-outs) and were excluded from all subsequent analyses.

### Comparisons Between Patients and HC

The remaining 26 patients who completed the study and the HC were not significantly different on sex distribution (50 vs. 44% females, respectively; χ^2^ = 0.18, *p* = 0.67), mean age (patients 52.1 ± 9.3 years, HC 48.2 ± 7.4 years; *t* = 1.66, *p* = 0.10), mean BMI (patients 28.0 ± 5.1, HC 26.5 ± 4.3; *t* = 1.14, *p* = 0.26), diagnosis of diabetes mellitus (patients 7.7%, HC 12%; Fisher's exact *p* = 0.67), median IL1β plasma concentration (patients baseline 0.28 pg/ml, HC 0.30 pg/ml; Mann–Whitney *z* = 1.05, *p* = 0.30) (Supplementary Figure 1A) and median IL1β lysate concentration (patients baseline 5.29 pg/ml, HC 6.56 pg/ml; Mann–Whitney *z* = 1.39, *p* = 0.16) (Supplementary Figure 1B). Spearman correlations of IL1β plasma and IL1β lysate concentrations were rho = 0.42, *p* = 0.03 in patients and rho = 0.30, *p* = 0.15 in HC. BMI had non-significant correlations with IL1β in plasma and lysates in both patients and HC.

### Correlation of Depression Severity With IL1β Concentration in Lysates and Plasma

Spearman correlations of patients' MADRS scores with IL1β lysate concentrations were non-significant both at T0 (rho = −0.20, *p* = 0.32) and T1 (rho = 0.29, *p* = 0.15). Similarly, non-significant Spearman correlations were recorded between MADRS scores and IL1β plasma concentrations both at T0 (rho = −0.29, *p* = 0.15) and T1 (rho = 0.06, *p* = 0.77).

### Comparisons Between Responders and Non-responders

Based on ΔMADRS(%), the 26 patients who completed the study were grouped into 17 responders and 9 non-responders. All patients were under medication at both time-points. As shown in Table 1, the two groups were not significantly different on medical comorbidity, number of previous antidepressant trials, baseline medication [types of psychotropics, antidepressant fluoxetine equivalent doses (46)], baseline MADRS scores, and most clinicodemographic characteristics, with the exception of a significantly lower baseline BMI for responders (*p* = 0.029).

**Table 1:** Clinicodemographic characteristics of study participants, IL1β concentrations, and their changes over time by response status.

		**Non-responders** **(***N*** = 9)**	**Responders** **(***N*** = 17)**	**Comparison** **(***P***-value)**
Sex (females)		7 (77.8)	6 (35.3)	0.10^a^
Age (years)		51.7 ± 6.1	52.3 ± 10.8	0.87^b^
Education (years)		12.3 ± 3.2	13.5 ± 4.0	0.47^*b*^
Living alone		2 (22.2)	2 (11.8)	0.59^a^
Employed		3 (33.3)	8 (47.1)	0.65^a^
Age at onset (years)		40.9 ± 11.6	41.2 ± 14.4	0.96^b^
Illness duration (years)		10.8 ± 11.8	11.1 ± 12.4	0.95^b^
MDEs lifetime		2 (2, 7)	2 (1, 3)	0.36^c^
Hospitalizations lifetime		2 (1, 3)	1 (1, 3)	0.72^c^
Suicide attempts lifetime		1 (0, 2)	1 (0, 1)	0.30^c^
Psychosis lifetime		1 (11.1)	7 (41.2)	0.19^a^
Psychiatric comorbidity lifetime		5 (55.6)	11 (64.7)	0.69^a^
CIRS items 1–13		8 (7, 9)	7 (4, 9)	0.10^c^
Diabetes mellitus		0 (0)	2 (11.8)	0.53^a^
Hypertension		5 (55.6)	8 (47.1)	1^a^
Dyslipidemia		3 (33.3)	7 (41.2)	1^a^
BMI (T = 0)		30.9 ± 6.1	26.4 ± 3.7	**0.029** ^**b**^
Previous AD trials		2 (2, 4)	2 (1.5, 3)	0.34^c^
Medication (T = 0)	SSRIs	6 (66.7)	11 (64.7)	1^a^
	SNRIs	3 (33.3)	3 (17.6)	0.63^a^
	Other ADs	1 (11.1)	8 (47.1)	0.10^a^
	APs	4 (44.4)	10 (58.8)	0.68^a^
	Lithium	0 (0)	2 (11.8)	0.53^a^
AD fluoxetine equivalent doses (mg/day)		59.6 ± 24.1	49.1 ± 17.1	0.22^b^
MADRS	T0	41.1 ± 6.9	40.9 ± 6.1	0.93^b^
	T1	27.9 ± 9.1	8.0 ± 5.0	**0.0001** ^**b**^
ΔMADRS(%)		31.6 ± 23.1	80.7 ± 11.1	**0.0001** ^**b**^
IL1β_lysates (pg/ml)	T0	4.6 (4.1, 5.4)	6.1 (4.3, 7.5)	0.11^3^/0.09^d^
	T1	6.6 (5.7, 7.7)	4.4 (3.5, 6.0)	0.07^3^/0.18^d^
IL1β_plasma (pg/ml)	T0	0.28 (0.22, 0.34)	0.28 (0.20, 0.34)	0.89^3^/0.94^d^
	T1	0.24 (0.22, 0.28)	0.18 (0.16, 0.32)	0.55^3^/0.77^d^
ΔIL1β_lysates(%)		−44.9 (−51.4, −26.0)	31.5 (0.69, 62.9)	**0.012**^2^/**0.016**^e^
ΔIL1β_plasma(%)		5.6 (−8.3, 16.7)	12.3 (−26.5, 26.5)	0.78^2^/0.84^e^

*AD, antidepressant; AP, antipsychotics; MDE, major depressive episode; SSRI, selective serotonin reuptake inhibitor*.

*ΔMADRS(%) = [MADRS (T0) – MADRS (T1)]/MADRS (T0) × 100, i.e., a positive ΔMADRS(%) denotes an improvement (decrease) of depression severity*.

*ΔIL1β(%) = [IL1β (T0) – IL1β(T1)]/IL1β (T0) × 100, i.e., a positive ΔIL1β (%) denotes that IL1β concentrations decreased*.

*Mean ± SD or median (25th, 75th percentiles) or N (%) is displayed. For IL1β concentrations and ΔIL1β(%) changes, p-values correspond to unadjusted/BMI-adjusted comparisons*.

a *Fisher's exact test*;

b *t-test*;

c *Mann–Whitney test*;

d *regression of log-transformed IL1β values on group adjusting for BMI*,

e *regression of ΔIL1β(%) on group adjusting for BMI*.

*Bold p < 0.05*.

**Figure 1:**
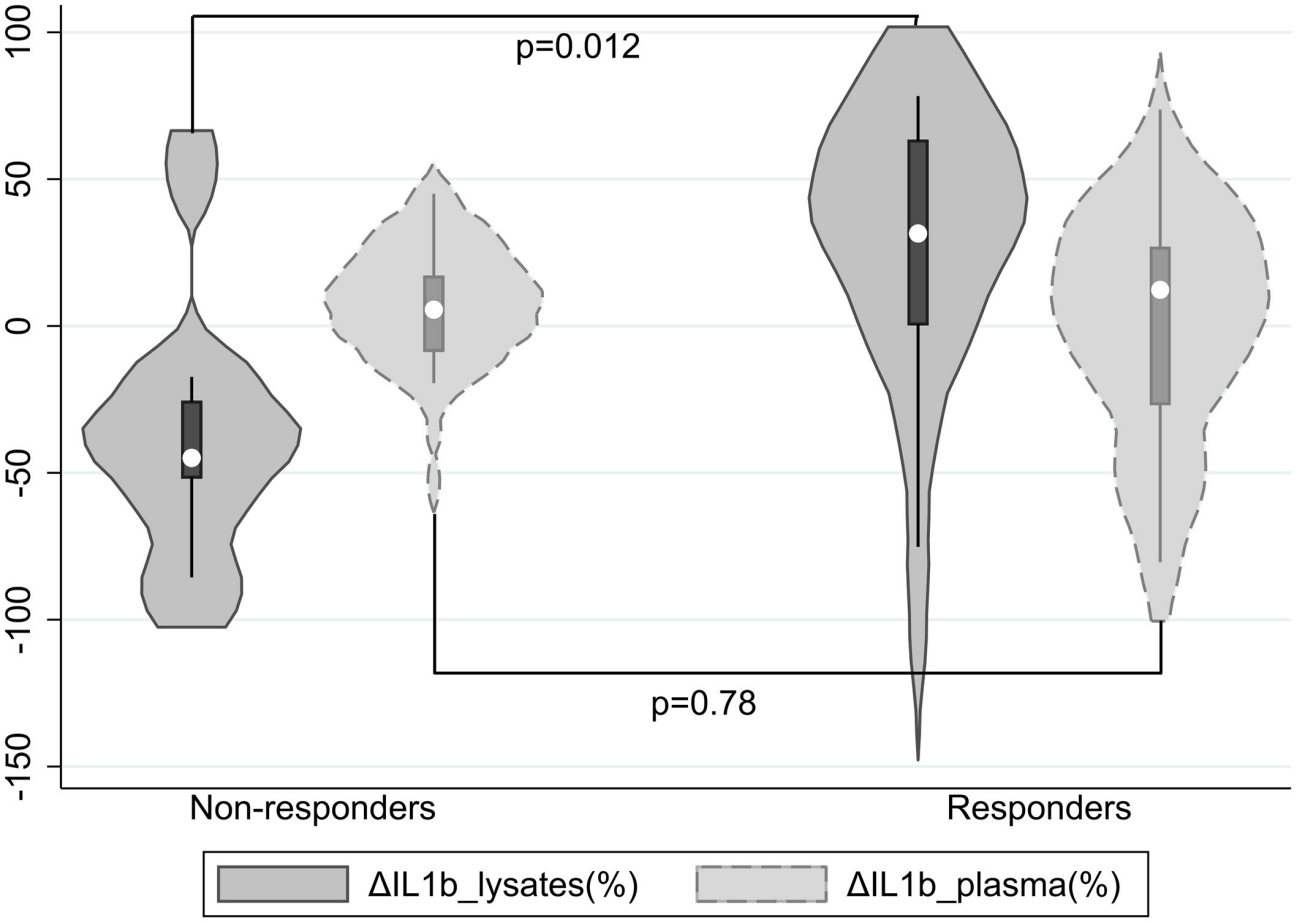
Violin plots of ΔIL1β_lysates(%) and ΔIL1β_plasma(%) by response status; a positive ΔIL1β(%) denotes that IL1β concentrations decreased.

Furthermore, the two groups did not significantly differ on IL1β plasma concentrations at T0 and T1 or their % change from baseline [ΔIL1β_plasma(%)]; results were not modified after adjusting for BMI. However, compared with non-responders, responders had suggestively lower IL1β lysate concentrations at T1 (*p* = 0.07) despite higher (though non-significantly) ones at T0 and a significantly greater % change of IL1β lysate concentrations from baseline [ΔIL1β_lysates(%)] (*p* = 0.012, Cohen's *d* = 1.13, 95% CI 0.25–1.98) (Table 1, Figure 1), which remained significant (*p* = 0.016) after adjusting for BMI.

Overall, ΔIL1β_lysates(%) and ΔIL1β_plasma(%) in the total sample were minute (medians 6.4 and 11.2%, respectively) and their correlation was non-significant (rho = 0.01, *p* = 0.96).

### Longitudinal Changes of IL1β Concentrations by Response Status

We investigated changes of IL1β lysate concentrations over time in the total sample and by response status in linear mixed models with ln(IL1β_lysates) as dependent variable and subject as random effect. The overall treatment (time) effect was non-significant (*p* = 0.13). When response group, time, and their interaction were included as fixed effects, the response group by time interaction term was significant (*p* = 0.005) (Supplementary Figure 2A), with non-responders displaying a non-significant increase in IL1β_lysates over time (*p* = 0.21) compared with responders showing a significant decrease (*p* = 0.002) (Supplementary Figure 2B). The interaction remained significant (*p* = 0.005) when BMI was also added as a fixed covariate.

Similar linear mixed models were built with ln(IL1β_plasma) as dependent variable. The overall time effect in the total sample was non-significant (*p* = 0.37). The response group by time interaction term was also non-significant (*p* = 0.96) (Supplementary Figure 3), with both response groups showing small non-significant decreases in IL1β_plasma over time. The interaction remained non-significant (*p* = 0.96) after adjusting for BMI.

### Predicting Response Status From BMI, Baseline IL1β Levels, or ΔIL1β(%) in Lysates or Plasma

Response status was significantly predicted by lower BMI: OR = 0.82, *p* = 0.048, and Nagelkerke pseudo-*R*^2^ = 0.24. Response status was not significantly predicted in logistic regression models by baseline IL1β levels in either lysates (*p* = 0.19) or plasma (*p* = 0.99); results were not modified after adjusting for BMI.

Response status was significantly predicted by ΔIL1β_lysates(%): OR = 1.02, *p* = 0.025, Nagelkerke pseudo-*R*^2^ = 0.31. This effect remained significant (OR = 1.02, *p* = 0.032) after adjusting for BMI (OR = 0.80, *p* = 0.067). In contrast, response status was not significantly predicted by ΔIL1β_plasma(%) before (OR = 1.00, *p* = 0.77) or after (OR = 1.00, *p* = 0.84) adjusting for BMI.

### ROC Curve Analyses and Classification

**Figure 2:**
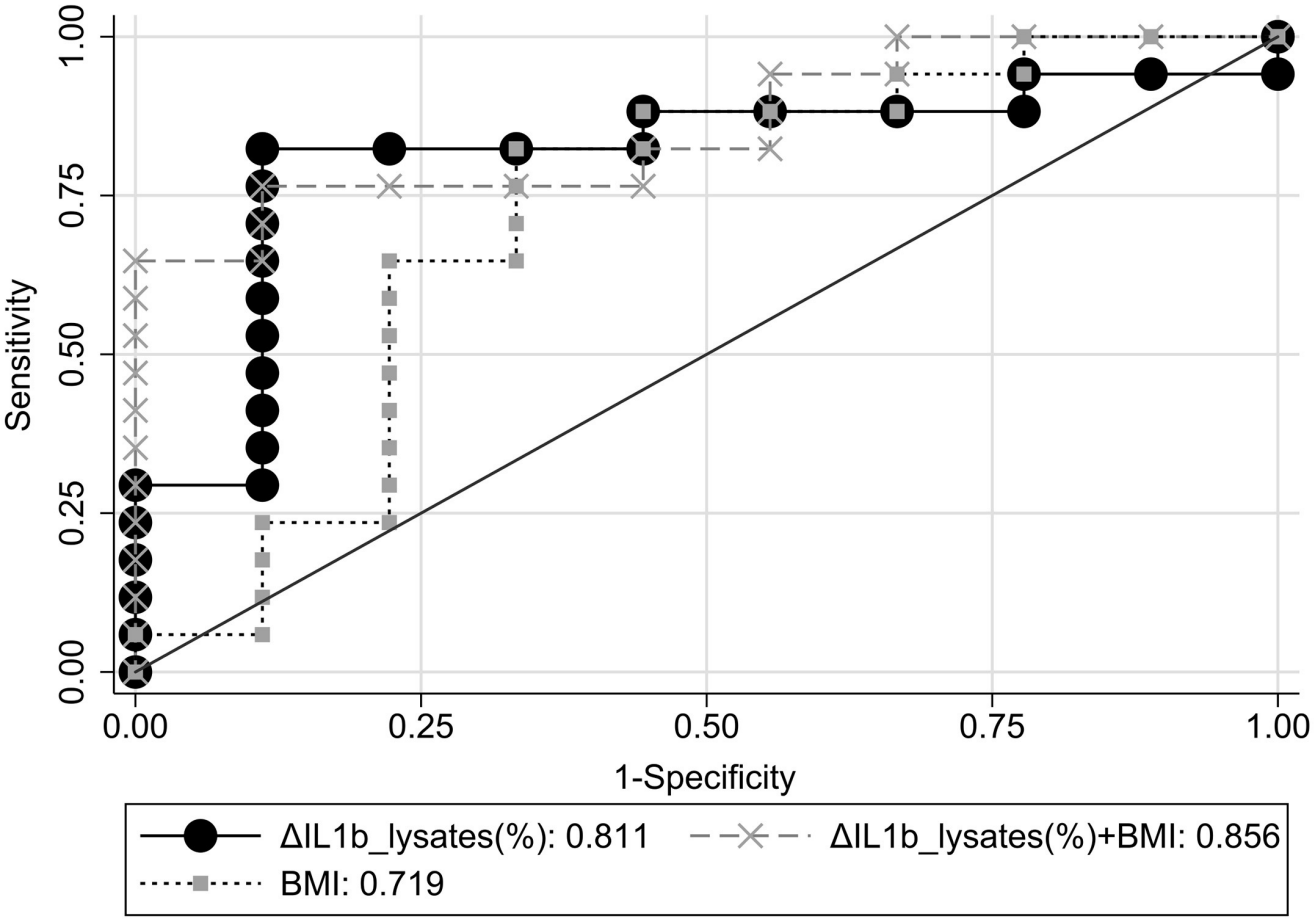
Comparative ROC curve analyses for response status using ΔIL1β_lysates(%), BMI, or ΔIL1β_lysates(%)+BMI combination as classifiers.

ROC curve analyses with response status as reference variable were performed using ΔIL1β_lysates(%), BMI, and ΔIL1β_lysates(%) combined with BMI or ΔIL1β_plasma(%) as classifiers (Supplementary Table 1). The areas under the ROC curves (AUC) and their 95% CI were calculated and compared among them (Figure 2). ΔIL1β_plasma(%) had AUC = 0.52, i.e., achieved chance-level classification and worse than ΔIL1β_lysates(%) or the combined classifier [ΔIL1β_lysates(%)+BMI] (*p* = 0.055 and *p* = 0.006, respectively). The combined classifier was nominally better than ΔIL1β_lysates(%) alone but not to a significant extent (*p* = 0.59). These two classifiers were the only ones with an AUC ≥ 0.8 and therefore of potential clinical utility.

The classification performance achieved at the optimal probability threshold by the two best classifiers, i.e., ΔIL1β_lysates(%) and the combined classifier, are compared in Table 2. Accuracy in our case–control dataset was 84.6 and 80.8%, respectively. Overall, ΔIL1β_lysates(%) had superior diagnostic metrics; the optimal probability threshold corresponded to a ΔIL1β_lysates(%) cut-off of ≥-2.42%. However, PPV, NPV, and accuracy are known to vary with the prevalence (p) of the condition under study as follows: PPV=p^*^PLR/[1+p^*^(PLR−1)], NPV=(1–p)^*^(1/NLR)/[1+(1–p)^*^(1/NLR−1)], and accuracy=Sn^*^p+Sp^*^(1–p) (47). Therefore, we adjusted our estimates with the aforementioned formulae to obtain extrapolations in the overall MDD population based on assumed response rates of *p* = 33, 50, or 67% (2, 3). Extrapolated accuracy was highest for ΔIL1β_lysates(%) at all assumed response rates (Table 2). Therefore, ΔIL1β_lysates(%) was the optimal and most parsimonious classifier for predicting antidepressant response in the context of this study.

**Table 2:** Classification performance of ΔIL1β_lysates(%) and ΔIL1β_lysates(%)+BMI combination at the optimal probability threshold.

**True**	**Classified as**							
	***ΔIL1β_lysates(%)***				***ΔIL1β_lysates(%)+BMI***			
	**R (+)**		**NR (–)**		**R (+)**		**NR (–)**	
R (+) 17	14		3		13		4	
NR (–) 9	1		8		1		8	
Totals 26	15		11		14		12	
**Optimal probability threshold**	≥0.678				≥0.728			
	[ΔIL1β_lysates(%) ≥−2.42%]							
**Diagnostic metrics**	**Sample**	**MDD^#^**			**Sample**	**MDD^#^**		
		**33%**	**50%**	**67%**		**33%**	**50%**	**67%**
Sn	82.4%				76.5%			
Sp	88.9%				88.9%			
PLR	7.41				6.88			
NLR	0.20				0.26			
PPV	93.3%	78.5%	88.1%	93.8%	92.9%	77.2%	87.3%	93.3%
NPV	72.7%	91.1%	83.4%	71.3%	66.7%	88.5%	79.1%	65.0%
CCR	84.6%	86.7%	85.6%	84.5%	80.8%	84.8%	82.7%	80.6%

*R (+), responder; NR (–), non-responder*.

# *MDD = overall MDD population extrapolation assuming probability of response p = 33%, 50%, 67%*.

*Sn, sensitivity; Sp, specificity; PLR, positive likelihood ratio; NLR, negative likelihood ratio; PPV, positive predictive value; NPV, negative predictive value; CCR, correct classification rate (accuracy)*.

### Power Analyses

For the comparisons between depressed patients (completers) and HC on IL1β (plasma or lysates) concentrations, the minimum effect size detectable with adequate power (0.80) was 0.80 [large by Cohen's rules of thumb (48)]. For the comparisons between responders and non-responders on plasma or lysates ΔIL1β(%), which were based on *t*-tests for three different response rates, the minimum adequately detectable effect size was 1.15 assuming a response rate of 50% and 1.20 assuming response rates of 33 or 67%.

## Discussion

The quest for biological markers of depression and antidepressant response has been a long-standing research priority. Biomarkers can be classified based on their utility: they can help predict risk of disease onset; establish diagnosis or stratify according to disease subtypes, severity, or staging; predict treatment response or overall prognosis; and longitudinally monitor treatment progress during follow-up (49). To achieve clinical utility, a biomarker should be highly accurate, disease specific, reproducible, and easily applicable (50). Biomarkers investigated are related to inflammatory, neurotransmitter, neuroendocrine, neurotrophic, oxidative/nitrosative stress, and metabolic biological systems, assessed at the level of genetics, epigenetics, and gene expression (51) or at the peripheral level (proteomics, metabolomics) (52).

IL1β belongs to the IL1 superfamily of cytokines and receptors, which is unique in immunology because it is primarily associated with innate immunity more than any other cytokine family (17). The role of IL1 has been extensively studied both in inflammation and in cell-mediated immunity, which are regarded as plausible pathomechanisms of depression (53–55). Our study focused on IL1β and its novelty lies in the simultaneous detection of the cytokine in plasma samples and in mononuclear cell lysates of healthy controls and depressed patients over the course of their treatment. An 8-week time-frame was chosen to assess response as 56% of those who responded in step 1 of the STAR^*^D study did so only at or after 8 weeks of treatment (4).

We found that baseline IL1β concentrations derived from either plasma or lysates could not efficiently discriminate between depressed patients and healthy controls. A meta-analysis found circulating IL1β levels higher in MDD patients compared with controls only in high-quality studies (28) while other meta-analyses found the difference to be non-significant although with a large heterogeneity, partly explained by MDD patients' BMI (10, 29).

Depression severity was not significantly correlated with IL1β levels both in lysates and plasma at both T0 and T1. Serum IL1β levels strongly correlated with depression severity in late-life depression (56). On the other hand, other studies found no significant associations between plasma or serum concentrations of several cytokines (including IL1β) and depression severity scores (57, 58).

Moreover, baseline IL1β concentrations derived from either plasma or lysates could not efficiently discriminate between future responders and non-responders and did not significantly predict response status; all results were not altered after adjusting for BMI, which was significantly lower in responders. These findings are in accordance with a recent meta-analysis (12).

The overall treatment (time) effect for IL1β derived from both lysates and plasma was found non-significant, resulting in their % change from baseline [ΔIL1β(%)] being minute in the total sample. Levels of circulating IL1β decreased after antidepressant treatment in an earlier meta-analysis (13), but more recent ones have not confirmed an overall effect of antidepressants when response status is not considered (14, 16). Yet, treatment with selective serotonin reuptake inhibitors, specifically, has been more consistently shown to decrease IL1β levels (13–15).

The most important finding of our study was that, when response status was taken into account, both response groups showed similar, small, non-significant decreases in plasma IL1β levels over time, while IL1β in lysates displayed contrasting trajectories between response groups over time, resulting in a significant response group by time interaction effect. In addition, ΔIL1β_lysates(%) significantly predicted response status, and this effect was robust to adjusting for BMI. Recent meta-analyses found no significant differences between responders and non-responders in the change of plasma or serum IL1β levels over the course of treatment (12, 16). Therefore, our results suggest that, unlike previous studies based solely on plasma or serum measurements, IL1β lysates assays may provide a promising, more sensitive longitudinal marker reflecting response to antidepressants. The biological significance of this finding may rely on the hypothesis that the pathophysiology of MDD is more related to activated, IL1β-enriched peripheral monocytes infiltrating the brain and interacting with microglial cells (30) than circulating IL1β (produced by peripheral monocytes and numerous other cell types) crossing the blood–brain barrier (22). Therefore, our results are more comparable with findings in transcriptional studies measuring IL1β mRNA in mononuclear lysates of depressed patients over treatment (31, 34).

Whether ΔIL1β_lysates(%) might serve as a surrogate endpoint predicting and temporally preceding the clinical endpoint of antidepressant response should be explored in future rigorous prospective studies including several time-points during follow-up (59). The clinical utility of such a surrogate endpoint would be to avoid frequent or untimely switching of antidepressants and finally improve outcomes (60).

Since IL1β strongly correlates with BMI, insulin resistance, and chronic inflammation (61, 62) and is implicated in various cardiometabolic conditions, such as obesity (63), diabetes (35, 36), and atherosclerosis (64, 65), these factors should be taken into consideration when proinflammatory cytokines are studied. The two response groups in our sample had no significant differences on physical health and metabolic indices, except for a lower baseline BMI in future responders, in line with previous evidence (66). Yet, correlations of BMI with IL1β in plasma and lysates were non-significant and the effect of ΔIL1β_lysates(%) on antidepressant response proved robust to adjusting for BMI.

ROC analyses identified two classifiers of antidepressant response with potential clinical utility: ΔIL1β_lysates(%) and ΔIL1β_lysates(%) combined with BMI. The latter had a nominally (though not significantly) larger AUC, but it achieved lower accuracy and worse overall diagnostic metrics at the optimal probability threshold both in our sample and when extrapolated to the general MDD population over a wide range of assumed treatment response rates. Therefore, ΔIL1β_lysates(%) proved to be the optimal and most parsimonious classifier for predicting antidepressant response with an accuracy of 85%. Using the cut-off of ≥-2.42% roughly suggests that depressed patients who display decreases in IL1β sampled from lysates over the course of treatment should probably be classified as responders.

Our study has potential limitations. In particular, our relatively small sample size limited the study's power to detect effects of small and moderate size, especially across response groups. In addition, the use of only two time-points did not allow us to assess whether an early surrogate biomarker endpoint (e.g., at 4 weeks) efficiently predicts a subsequent clinical endpoint (e.g., at 8 weeks), which would be of great clinical interest and needs to be investigated in future studies. Finally, our findings await replication in future independent samples.

In conclusion, the results of this comparative pilot study suggest that antidepressant responders and non-responders displayed contrasting IL1β trajectories in lysates but not in plasma assays. Therefore, percent change of IL1β lysate concentrations from baseline predicted response with an accuracy of 85%, arising as a potential longitudinal surrogate marker of antidepressant response warranting further investigation.

## Data Availability Statement

The raw data supporting the conclusions of this article will be made available by the authors, without undue reservation.

## Ethics Statement

The studies involving human participants were reviewed and approved by Attikon Hospital Research Ethics Committee. The patients/participants provided their written informed consent to participate in this study.

## Author Contributions

PF contributed to study design, was responsible for the recruitment of participants, supervised clinical assessments, performed the statistical analyses, and drafted the article. EM contributed to study design, performed the biological assays, and drafted the article. AA performed the clinical assessment of participants. AS and NS critically revised the article and contributed comments and suggestions. PM formulated the original research hypothesis and contributed to study design, secured funding for the biological assays, and critically revised the article. All authors contributed to the article and approved the submitted version.

## Funding

Funding for the biological assays included in this study was provided by a block grant of the National and Kapodistrian University of Athens, Faculty of Medicine to the Department of Clinical Biochemistry.

## Conflict of Interest

The authors declare that the research was conducted in the absence of any commercial or financial relationships that could be construed as a potential conflict of interest.

## Publisher's Note

All claims expressed in this article are solely those of the authors and do not necessarily represent those of their affiliated organizations, or those of the publisher, the editors and the reviewers. Any product that may be evaluated in this article, or claim that may be made by its manufacturer, is not guaranteed or endorsed by the publisher.
