# Supplementary material for: Interleukin-1 Beta in Peripheral Blood Mononuclear Cell Lysates as a Longitudinal Biomarker of Response to Antidepressants: A Pilot Study
Source: Front Psychiatry. 2021 Dec 24;12:801738. doi: 10.3389/fpsyt.2021.801738 (PMC8738167; doi:10.3389/fpsyt.2021.801738)
Supplement: Supplementary file 1 [file Data_Sheet_1.docx]

**Suppl. Figure 1.** Violin plots of IL1b_plasma (a) and IL1b_lysates (b) concentrations (pg/mL) by diagnosis (depressed patients vs healthy controls).

**(a)**

**(b)**

**Suppl. Figure 2.** Response group by time interaction plot of (ln)IL1b_lysates concentration (a) and time (T1-T0) contrasts by response status (b).

**(a)**

**(b)**

**Suppl. Figure 3.** Response group by time interaction plot of (ln)IL1b_plasma concentration.
