# Supplementary material for: Interleukin-1 Beta in Peripheral Blood Mononuclear Cell Lysates as a Longitudinal Biomarker of Response to Antidepressants: A Pilot Study
Source: Front Psychiatry. 2021 Dec 24;12:801738. doi: 10.3389/fpsyt.2021.801738 (PMC8738167; doi:10.3389/fpsyt.2021.801738)
Supplement: Supplementary file 2 [file Table_1.DOCX]

**Suppl. Table 1.** ROC curve analyses for response status using ΔIL1b_lysates(%), BMI, ΔIL1b_lysates(%)+BMI combination or ΔIL1b_plasma(%) as classifiers.

|  | Classifier | AUC | SE | 95% CI | Comparison (p-value) | | |
| --- | --- | --- | --- | --- | --- | --- | --- |
|  |  |  |  |  | vs 2 | vs 3 | vs 4 |
| 1 | ΔIL1b_lysates(%) | 0.811 | 0.096 | 0.623 - 0.998 | 0.592 | 0.586 | 0.055 |
| 2 | BMI | 0.719 | 0.124 | 0.476 - 0.962 |  | 0.178 | 0.139 |
| 3 | ΔIL1b_lysates(%)+BMI | 0.856 | 0.073 | 0.713 – 1.000 |  |  | 0.006 |
| 4 | ΔIL1b_plasma(%) | 0.516 | 0.121 | 0.278 - 0.754 |  | | |

AUC= area under the ROC curve; SE= standard error of AUC
